# Lecture on the Morphology of the Blood in Syphilis

**Published:** 1879-11

**Authors:** Ephraim Cutter

**Affiliations:** Boston.


### ARTICLE IV.

### *Lecture on the Morphology of the Blood in Syphilis.*

BY EPHRAIM CUTTER, M. D., BOSTON.

Delivered before the Maryland and District of Columbia Dental Association, September, 1878.

The word "Morphology; Morphos—Form and Logos—Discourse; is applied to the ideal forms of the parts or organs in the structure of plants and animals—their varieties, homologies and metamorphoses." There seems to be no good reason why in so real a matter as our subject, the ideal use of this term should not be extended to its practical employment in order to denote the description of the forms or substances found in the blood and tissues of persons suffering hereditarily or by inoculation with syphilis. We define blood as a fluid found in the vascular system of the human body of a very complex constitution, which we will divide into

1.—Fluid or plasma.
2.—White corpuscles.
3.—Colored corpuscles.
4.—Foreign substances that possess morphological characters.

Position of the writer; after more than ten years experience he comes as a witness—not as the discoverer—not polemically, but as sincerely believing that what he states is true and valuable to the medical profession. Ridicule or even contempt cannot annul history. I do not say that I make no mistakes, but I will not apologise for acting up to my convictions of duty.

In the *American Journal of Medical Science* for January, 1868, appeared a description of two new algoid vegetations, one of which appears to be the specific cause of syphilis, etc., by Dr. J. H. Salisbury, M. D. *Chief points* —Syphilis first attacks the connective tissue at the point of inoculation and next the lymphatic glands, after the primary sore or sores have healed. The specific cause may remain apparently dormant in the system for a period varying from a few days to months and even years; it then becomes evidenced by blotches and hard swellings in the periosteum. Secondary: Thence it may spread its lesion to bone and cartilage "Tertiary." The author asserts that his results have been arrived at by long continued, patient and careful labor, and that it is possible that he over estimates his results. He began to figure and describe every new body and circumstance in Micrology in 1849. Eleven years he examined the pus from chancres and made no progress. The only thing he found foreign were small copper colored minute globular bodies. But on dissecting out the bed of chancres, filaments running in all directions were soon discovered. Up to 1868 one hundred primary sores had uniformly showed this vegetation. He discovered this vegetation in the blood wherever the disease had become constitutional. On its presence or absence in the blood he has based a sure guide for treatment, judging from his practical experience. He says: "Under favorable states of the system the tendency seems to be for the vegetation to gradually lessen, and probably in some few instances it may eventually disappear entirely. It may be transmitted during the secondary and tertiary stages under the proper conditions without producing the primary disease. I have noticed many instances in which the father, having had the disease previous to marriage and when the poison was not entirely eliminated, even though no outward manifestations of the disease had shown itself after marriage; this vegetation was transmitted to, and found in the blood of the wife and children many years after. In many cases of this kind this vegetation produces no visible impression upon the systems to which it is transfered, while upon others it produces more or less marked constitutional disturbance."

The following is a condensed description of the vegetation: Genus crypta—Minute, transparent, highly refractive, copper colored algoid filaments, which develope in living tissues from spores. Crypta syphilitica—Salisbury—a homogeneous filament with extremities obtusely rounded. The filaments are of such uniform structure throughout that no trace of transverse markings are visible save in their early stage of development. Neither can the contents be distinguished from the outside wall. The filaments are either straight, coiled, or arranged in curves; develope from spores active or inactive in the connective tissue, and may be transplanted from one individual to another by inoculation or by contact with mucous membranes. They are believed to produce the disease named syphilis.

History of this the contribution. It has been received with a total denial by some, while others have verified the observations and found them of great practical value. Among the latter I come. Lorstofer made some announcements about a syphilitic corpuscle found in the blood. But this was subsequent to 1868. His theory was not accepted. In my opinion he only had half the truth. Other diseases have enlarged white corpuscles. Salisbury pointed this out before he gave the name of Zymotosis Regularis to a vegetation he discovered in healthy blood (Microscopic Examinations of Blood, Moorehead, Bond & Co., N. Y., page 32.) Lostofer's method was open to the objection that the blood was to be kept sometime and a vegetation developed therefrom.

Salisbury's method depends on getting the blood from the capillaries as soon as possible, since the spores are perishable. Lorstofer does not allude to the copper color of the spores, inside of the white corpuscles, nor to the filaments in the blood and peripheral margins of chancres; nor to the copper colored spores in the pus; nor to the differential diagnosis from other diseases. Lorstofer was correct in the fact that the white corpuscles are enlarged in syphilis, but he should have insisted upon the copper colored spores contained within as a distinctive mark. The difficulty is that he did not take in the full scope of the biology of the vegetation. He deserves credit for the work he did and for opening up the field in the manner he did, though it was partially done.

The Salisbury position.—He finds in the blood of syphilis algoid forms of vegetable life, composed of copper colored spores and filaments with obtusely rounded ends. He finds them in the blood of parents, and in the blood of their children; thus showing a palpable hereditary taint. He finds the spores and filaments in the primary sores, and on the infected mucous surfaces. "It is possible that I overestimate what I have found, but I believe them to produce the disease called syphilis. Time and careful investigation can only determine the truth." This is not boastful language.

Before proceeding further, let us ask what are algae and what is algoid? Algae are plants that include the sea-weed and many fresh water plants. Pull in a Devil's Apron string from the beach and you have an algæ. Pick out a stone from a fresh water pool covered with green filaments and you have algæ. It may be the common œdogonium or spirogyra. There are over 12,000 species of algæ. Algæ are characterized by the production of oxygen gas from the carbonic acid gas found in the media in which they grow. They contain also chlorophyll and starch and protoplasm. They are parasitic on the human body. The *sarcina ventriculi* of Goodsir is an algæ. I have found it in the waters of Fresh Pond, Cambridge, Mass.; in the Cochituate, Boston; and Prof. Reinsch found it in the waters of the Lagoon between the steamboat landings at Oak Bluffs. Algæ have been found by Dr. Salisbury and by myself in the spleen of the Turtle. This vegetation was probably harmless. Algoid—like algæ.

To make this subject clear, we ask: What is a fungus?

Medicines sometimes grow mouldy and present flocks of matter floating in the substance of the liquid; these are fung. Under a 1-5th inch objective and an eyepiece it appears made up of threads or filaments called fungus or mycelial filaments. (Mukos meaning mushroom.) These mycelial filaments form the thallus or frond of the mushroom, corresponding to the trunk of trees; the other parts of the fungus is its fruit. This varies in form. In the yeast it is made up of large obovoid or globar compound bodies that seem ready to burst, and in fact do burst, discharging their contents—the spores—and scattering them about like seeds, when are ready for development if a suitable nidus be found to lie inactive waiting for favorable circumstances. The large bodies are called sporangia or spore cases. Fungi give off carbonic acid gas, have no starch, no chlorophyll, are protoplasmic. *Algæ* have the power of independent motions. There is one family called oscillatoriaceæ, as they oscillate backward and forward like the tail of an enraged cat. Sometimes advancing forwards and then going backwards—creeping like a worm. Bacterium and Vibriones form Genera in this family. 8000 species of fungi are described.

It is germain to ask to what extent do parasitic vegetations exist in the world? Prof. Reinsch answers this question as follows:

List of Parasitic Vegetations.

[A.]—Entophytic in the parenchyma of other plants,—*Algae* melanospermæ, Black Sea Weeds, Entonema, New Genus, Ectocarpeorum. All found in the thallus or frond of nearly all larger Floridae.

[B.]—Rhodospermæ, (Floridæ,) Red Seaweeds, Choreocolax, Entocolax, Syringocolax, fructification outside the infected plants arising from a stroma like hypo-thallus. Compare Reinsch's contributiones ad algologiam etc., illustrated in 12 plates. Found in the thallus of many other Floridæ, Polysiphonia, Plocanium, Alsidium, Hypnea, Ceramium, etc.

[C.]—Chlorospermeæ, Chroolepus and Muscicola, Reinsch contributiones, both in intercellular spaces and in cells of the leaves of Jungermania.

[D.]—Phycochromophycaceæ Algae, Nostoc and Polycoc cus in the Parenchyma of Liverwort, Marchantia and Conocephalus Scytonema observed in the Parenchyma of green house plants in the Botanical Institution of Leipsic. The filaments of Scytonema growing through the Stomata and expanding on the surface of the epidermis.

[E.]—Fung. Many of the Hyphomycetes and Coniomycetes. The Stroma of Potatoe Fungus. Grape Fungus. Mildew. Eryside Sper on the leaves and stems of Leguminosæ Gramineæ, etc.

[F.]—Saprolegniæ.

Several new Genera belonging to Chytridiaceæ inside the cells of Desmids consuming the whole contents of cells and killing them. New Genus of Chrytridiaceæ, forming three different cells. Male and female cells inside the Utricle of Saprolegnia. Very probably belong to the so-called "Asterospheres" observed in cells of nitella and Charsa.

EPIPHYTIC ON THE SURFACE OF OTHER PLANTS.

Melanospermæ and Rhodospermæ—more than 2000 species—most of those figured in the contribution are epiphytic. Chlorospermæ nearly 50 species.

VEGETABLE PARASITES GROWING ON ANIMALS.

[1.]—Plants living inside the organs. Leptothricæ and Fungoid filaments in blood—excretion of ears—Vibrionidæ, Globular Bacteria in urine.

[2.]—Algæ living on the surface of the animal body.

[A.]—Phycochromaceous Algæ.

Polycoccus and Micrococcus, skin disease caused by chignon. Rabela Algæ. European. Leptothrix in mouth.

[B.]—Saprolegniæ, Protoplasmic plants, Synchytrium, Allochytrium, Genus Novum, Reinsch. Achlya, Rhizogartes, Entocellular Saprolegniæ in desmids.

ARE ALL PARASITES NOCUOUS?

It would be a work of supererogation to ask this question were it not that the impression is abroad in the profession that allowing the existence of this algoid vegetation of Salisbury's it is harmless, as there are so many known vegetable parasites dwelling in and on their hosts as peaceful and advantageous guests. In reply to this question we say that while some parasites as the leptothrix buccalis for example are innocuous, there are some that victimize their hosts. Witness the muscardium on silk worms. The Botrytis infestans on potatoes. The puccinia on trees. The oidium on grape vines. Moreover this method of reasoning entirely ignores the facts of history in relation to poisoning people by eating mushrooms that are fungi.

Mr. Julius A. Palmer, Jr., of Boston, has made a study of edible and inedible fungi. He has collected accounts of sickness and death from eating fungi. It is to be hoped that he will be induced to give the profession the benefit of his studies.

We do not so reason in relation to trees and shrubs; because many fruits are innocuous, all are not. As huckleberries are wholesome it does not follow that all berries are. All things are classed as good or bad. It is idle then to reject Dr. Salisbury's contribution on the ground of the harmlessness of some parasitic vegetations.

Does the presence of these morphological elements remain in the blood during apparent good health? Yes it may. They may constitute a hereditary taint. Usually they disappear from the blood (pari passu) as the health improves.

How is the blood examined by the Salisbury plan?

Every thing depends on the proper method. The aim is to obtain the blood almost exactly as it is in the vascular system. This requires expedition and cleanliness.

[1.]—Things to be had. [A.]—The patient. [B.]—A lancet or scarificator. [C.]—A clean glass slide or an covered glass. [D.]—A positively good 1-5th inch objective and two inch eyepiece. [E.]—A good illuminator, Perkins & House safety lamp is the best I know of.

[2.]—Things to avoid. [A.]—Foreign substances, as dirt, etc., from the skin. [B.]—Too much blood. [C.]—Too little. The drop of blood should completely fill the space between the cover and slide and no more.

[3.]—Place of selection for the purpose of drawing blood: fore-arm, above the wrist.

[4.]—Time, patience, practice, expedition, careful observation is necessary.

[5.]—The observer should first learn how to distinguish white corpuscles from the red, and should study the forms and peculiarities of vegetations that are found on the body and about the body.

[6.]—A year of careful investigation will do more towards understanding this matter than any amount of writing or theorizing; that is, taking known syphilitics and examining their blood and chancres.

Position of the writer.—He testifies that the positions of Dr. Salisbury are verified, by st·dies that have ranged over ten years, and been prosecuted in private practice and in the following Hospitals: 1.—U. S. Naval, Brooklyn. 2.—U. S. Chelsea. 3.—State Almshouse, Tewksbury Mass. 4.—Bellevue, N. Y.

That he has found in syphilitic blood and in the beds of chancres, the copper-colored spores and filaments; enlarged white corpuscles in which the copper-colored spores have been detected, and also that he has been able to photograph these appearances with the highest power objectives, to wit: the 1–75th of Tolles, belonging to Dr. G. B. Harriman, dentist, of Boston, to whom the writer acknowledges his indebtedness for aid and the use of this instrument.

### Illustrations.

*The following Microphotographs were projected with a Marcy's Sciopticon. Owing to the great expense attendant on such a course we cannot publish the illustrations.*—Eds.

### Series 1.

An answer to the question what is a fungus?

Illustration 1.—Photograph of the apparatus. See *American Journal Sciences and Art*, August, 1879. Consisting

[A.] of a one inch base-board, 55 by 11 inches. [B.] A mirror about 1 foot square, mounted like a compass. [C.] A mounted Voigtlander's 18 inch focus camera objective 3½ inches in diameter. [D.] A first class Tolles. A stand with objectives to be named; no eyepiece; tube horizontal and opening into a camera whose plate was usually 30 inches from the object. The apparatus is simple, portable, and has been used at different stations. It is a modification of Dr. Woodward's apparatus. Acknowledgements of indebtedness have been made to him who, the speaker termed, "the father of modern microphotography." Allusion was made to a much simpler form of camera the writer had devised to be used with any microscope, as his contribution towards aiding the art.

[2.]—Microphotograph of the common yeast plant: 1–50th of an inch objective; 1300 diameters on the plate. This was to show the physical features of a fungus which mankind had known from time to time immemorial as of domestic importance and as the one fungus that had been studied more than any other fungus for the past 25 years. The illustration showed two sporangia of the Torula Cerevisiae; with their contained spores; protoplasm, and formed cell wall.

[3.] and [4.]—do.; 1–50th. Yeast and Barley Starch Grains. The interspaces displayed spores which, when photographed, were in protoplasmic motions, and caused blurred outlines. Some botanists say these spores have naught to do with the yeast; but belong to another fungus. While others say, they have seen the sporangia burst and discharge the same spores. The first call the spores bacteria.

[5.]—Showed the variations in the form of the sporangia.

[6.] and [7.]—Yeast plant enormously enlarged from a 1–50th objective. They show the apparently empty spaces common in white blood corpuscles and other protoplasmic bodies.

[8.] and [9.]—Starch grain apparently 1–3rd consumed in the act of being converted into alcohol, etc.

[10.]—Seven yeast plants photographed with the 1–75th objective. It shows more details of structure than the others did.

[11.]—Mitscherlich's drawing, showing the increase of yeast by budding. This process is doubted by some Botanists. It is difficult to explain this difference of opinion as to matters of fact; if such is the case when biological phenomena are patent and open to the eye, shall we wonder that honest differences shall also exist when the facts are occult and obscure?

[12.] and [13.]—1–5th objective. Filaments from the surface of yeast that had become mouldy. They are called mycelial from mukos, mushroom. Some say they have nothing to do with the yeast plants, others declare they come from the yeast. Still they are examples of the part that is analagous to the trunk of phanerogams.

[14.]—1–10th 4 system Tolles objective. Sewer-gas fermentation vegetation. A contribution to the physical demonstration of what may be nocous in the atmosphere of sewers.

[15.]—Vegetation found in rancid butter. A fungus growing in the parenchyma of an organic animal substance of far greater density than the blood.

[16.]—Diphtheritic membrane from the uvula of the daughter of the speaker, who died July 1st, 1874; kept in strongest carbolic acid till January, 1878. A portion thrown up with the helio-microscope showed multitudes of spores and filaments in active motion.

[17.]—Vaccine virus.

*Algae* form another class of parasitic plants.

[18.]—Leptothrix buccalis. Innocent.

[19.]—An algae found growing in the substance of a Key West sea weed. Innocent. 1–5th objective. From Prof. Reinsch.

[20.]—Desmid from Cochituate water; 1–800 inch long. Inside a curious development which Prof. Reinsch says is a growth resembling those found by Dr. Salisbury in the white blood corpuscles of syphilitic blood.

[21.]—Plate from Prof. Reinsch, showing many algae parasitic in other algae, but nocent and killing their host. These show that Salisbury had authority to sustain his positions.

DIVISION 2.

To answer the question, "What is the morphology of healthy blood."

1.—Human blood. 2.—Rat. 3.—Ox. 4.—Horse 5—Dog. 6.—Sheep. 7.—Trout. 8.—Frog. Taken with the 1–50th objective; at the same distance. Interesting in a medical point of view, and support the dicta of Woodward that no life should be taken on blood evidence alone. 9.—Human, 1–75 inch. 10.—Bluebird's blood, 1–16th inch objective. 11.—An example of healthy blood, outlines clear and clean cut; segregation distinct; interspaces clear and free from foreign substances. 12.—Healthy white corpuscle; 1–50th; no appearance of ento-phytal growths.

SERIES 3.

Morphology of syphilitic blood:

1.—Spore and spore collects; in the original they were copper colored.

2.—Do. with some fat globules from the areolar tissue of the skin.

3.—Four white corpuscles, enormous in size; distended with ento-phytal growths, called the syphilitic vegetation.

4.—1–50th, white corpuscle with marked ento-phytal growths; spores and red corpuscles.

5.—1–75th inch objective; white corpuscle with inside growths, one spore very much enlarged.

6.—Same objective, white corpuscle in which are several spores that were copper colored in the original.

7 and 8.—Two enlarged and over-distended white corpuscles with peculiar phases that cannot well be described.

9.—1–50th, white corpuscle which Prof. Reinsch declared morphologically identical with some ento-phyte he had discovered in algæ and described in a work published in

Germany. Later he wrote: "I see as botanist in this diseased blood nothing but a peculiar case of ento-cellular vegetable parasitism." Prof. Reinsch is the leading agologist in the world. His utterances sustain Dr. Salisbury.

10.—Algoid filament in syphilitic blood.

11.—A bent, obtusely ended filament, the bluntness is marked.

12.—Another.

13.—For comparison, spores of the pre-tubercular state usually present one year before the organic lung disease; not copper colored in the original.

14.—Mycelial filament; consumptive blood.

15.—1-75th objective. Three white corpuscles distended and enlarged by ento-phytal growths. These were introduced to show that there are other complaints in which there are spores--filaments and enlarged corpuscles—but that the copper color is the distinguishing characteristic of syphilis.

Series 4.

1. Hair in blood. 2. Cotton fiber. 3. Dust from furniture. 4. Dust from the skin of a patient. 5. Lard. 6. Soap. These may be sources of error.

Resume. Dr. Salisbury believes the peculiar contagion of syphilis to consist in a cryptogamic vegetation he calls crypta syphilitica he finds in the blood in chancres and in the mucous membranes of syphilides, and gives drawings of the same.

Second, the speaker has verified this discovery by ten years experience.

Third, he endeavors to give the evidence. A. By showing what is the morphology of a fungus. B. Of an algae. C. Of healthy blood. D. Of syphilitic blood. E. Of consumptive blood. F. Sources of error.

In these demonstrations he has shown microphotographs never before taken and has used the best instruments of precision known to modern art.

